# The change and effect of endothelial progenitor cells in pig with multiple organ dysfunction syndromes

**DOI:** 10.1186/cc7968

**Published:** 2009-07-15

**Authors:** Tian Hang Luo, Yao Wang, Zheng Mao Lu, Hong Zhou, Xu Chao Xue, Jian Wei Bi, Li Ye Ma, Guo En Fang

**Affiliations:** 1 Department of General Surgery, Changhai Hospital, The Second Military Medical University, Xiangyin Road, Shanghai 200433, PR China

## Abstract

**Introduction:**

The dysfunction and decrease of endothelial progenitor cells (EPCs) may play a very important role in the initiation of organ dysfunction caused by trauma or severe sepsis. We aim to measure the number and function of EPCs in the progression of multiple organ dysfunction syndromes (MODS) caused by severe sepsis, which may help to understand the pathogenesis of MODS by the changing of EPCs.

**Methods:**

A total of 40 pigs were randomly divided into two groups, which were subjected to hemorrhagic shock, resuscitation and endotoxemia (experimental group, n = 20) or acted as a control (control group, n = 20). The number and function of EPCs including adhesive, migratory and angiogenesis capacities were analyzed at different times in both groups.

**Results:**

All the animals in the experimental group developed MODS (100%) and 17 of 20 animals (85%) died due to MODS; the incidence of MODS and death of the animals in the control group were 0% (*P *< 0.01). The number, migratory and adhesive capacities of EPCs decreased sharply in the animals of the experimental group corresponding to the increasing severities of MODS, but the angiogenesis function increased gradually until death. The decrease in function of EPCs preceded the decrease in number of EPCs. The decrease in number and function of EPCs occurred prior to the occurrence of MODS.

**Conclusions:**

For the first time, it was observed that the number and function of EPCs decreased sharply in the progression of MODS and that it was prior to the occurrence of MODS. The decrease in number and function of EPCs may be one of the main pathogenic factors of MODS.

## Introduction

The endothelial cells (ECs) play a pivotal role in the progression of multiple organ dysfunction syndromes (MODS) caused by trauma or severe sepsis; the ECs are not only the participant of inflammatory reaction, but also the first damaged target cells. The dysfunction of ECs is a critical event in the initiation of organ dysfunction [1].

Recent studies suggested that the injured ECs could be regenerated by circulating bone marrow-derived progenitor cells called endothelial progenitor cells (EPCs), which had capabilities to mobilize from bone marrow with homing to foci of injuries and to differentiate into mature ECs to ameliorate the dysfunction of ischemic organs caused by trauma or severe sepsis. This procession was possibly induced and modulated by vasculogenesis and angiogenesis in areas with reduced oxygen and circulation supply or by stimulating the re-endothelialization of injured circulation vessels [2,3]. The impairment of EPCs caused by severe inflammation may therefore contribute to the progression of multiple organ dysfunction.

Following the first description of isolation of putative EPCs for angiogenesis by Asahara and colleagues in 1997 [4], increasing evidence indicated that bone marrow-derived circulating EPCs were involved in the process of neovascularization. EPCs were considered to originate from hematopoietic stem cells, which are positive for CD133 and the vascular endothelial growth factor receptor-2 (KDR) in the early stage.

Several lines of evidence now suggest that the ischemia of organs after trauma or severe sepsis would be the most important pathogenesis of MODS [5,6]. We therefore hypothesized that the number and function of circulating EPCs released from bone marrow would decrease sharply after major trauma, which would serve as early target cells in pathogenesis of MODS. The regularity of the number and function of EPCs in MODS, however, remained unclear. To verify our hypotheses, we set up an animal model of MODS and serially sampled blood and bone marrow from the model at the 24th hour before operation and at the 12th, 24th, 72nd, 96th, 144th and 168th hour after severe trauma to observe the dynamic variation of bone marrow and circulating EPCs in the various stages of MODS caused by two-hit injuries, including the number and function of EPCs.

## Materials and methods

### Animals

All experiments were performed in accordance with the China legislation on protection of animals and the 1996 National Institutes of Health *Guide for the Care and Use of Laboratory Animals *[7]. A total of 40 domestic male pigs with a body weight of 20 to 25 kg (22.41 ± 1.33 kg) were used for the present study. The animals were kept at 20 to 25°C all of the time, with daylight and free access to tap water and standard daily food. One day before the experiments, the animals were kept fasting overnight with free access to water. Forty pigs were divided randomly into two groups, which were subjected to hemorrhagic shock + resuscitation + endotoxemia (experimental group, n = 20) and the control group (n = 20).

### Anesthesia and positioning

After an intramuscular injection of 15 mg/kg ketamin hydrochloride (Pfizer, Karlsruhe, Germany), 0.4 mg/kg diazepam (Sunrise, Shanghai, China) and 0.02 mg/kg atropine (Braun, Melsungen, Germany) for premedication, general anesthesia was induced by intravenous injection of 1 mg/kg etomidate (Braun). Anesthesia was maintained by continuous intravenous injection administration of ketamin hydrochloride (5 to 10 mg/kg/hour; Pfizer) and diazepam (0.1 to 0.2 mg/kg/hour; Sunrise). Oral intubation was performed (7.5 ET Tube; Bezer, Shanghai, China) and the animals were mechanically ventilated (Evita, Dräger, Lübeck, Germany), volume cycled with a tidal volume of 10 ml/kg. The respiratory frequency was adjusted to maintain the peak inspiratory pressure below 30 mmHg, and an inspiratory oxygen concentration of 30% with a positive end-expiratory pressure of 2 cmH_2_O and an inspiratory/expiratory ratio of 1:2 were used. Cardiac and respiratory parameters were monitored throughout the procedures. The animals were positioned in the lateral decubitus position, alternating their right side and left side for bilateral access. Upon completion of the experiment, the survival animals were euthanized by overdosed intravenous injections of pentobarbital sodium (Tianyi, Xian, China).

### Operation

All of the experimental animals in the two groups underwent the same operation under aseptic conditions. Individual anatomical landmarks were marked on the animal's skin, including the cartilage thyroidea, the articulatio sternoclavicularis, the acromion, the inferior scapular angle, the pubic symphysis and the anterior superior iliac spine. First of all, the arteria carotis interna was dissected and intubated with a 12 G retention catheter (Arrow, Leeds, UK) to monitor the arterial blood pressure. The left femoral artery and femoral vein were then dissected and intubated with an 8 F Swan-Ganz catheter (Arrow) into the femoral vein to monitor the pulmonary arterial pressure, the pulmonary arterial wedge pressure, the ventricular stroke output and the central venous pressure. A retention catheter was intubated into the right femoral artery for exsanguination and all catheters were fixed. The skin of the animals was then sutured.

### Hemorrhagic shock, resuscitation and endotoxemia

The animals of the experimental group underwent hemorrhagic shock, resuscitation and endotoxemia after the operation. The hemorrhagic shock model was not induced by a modified Wigger's procedure until the animal's general condition was stable after operation. Hemorrhagic shock was produced for a period of 120 minutes by blood-letting via the femoral artery until the mean arterial blood pressure (MAP) was 6.7 ± 0.67 kPa (50 ± 5 mmHg) in 30 minutes. We then transfused 60% of the lost blood and lactated Ringer's solution (Otsuica, Tianjing, China), which was twice as much as the lost blood in 60 minutes. The MAP must reach over 80% of the MAP before hemorrhagic shock. The experimental pigs were intravenously injected with 0.5 mg/kg lipopolysaccharide of coli bacillus (E. colO_111_B_4_; Sigma, St Louis, Missouri, USA) 12 hours after the resuscitation for a period of 24 hours.

### Organ function monitoring and supporting

The experimental animals were given electrocardiographic monitoring and the observed indexes included the MAP, the breathing rate, the heart rate, the central venous pressure, the pulmonary arterial pressure, the pulmonary arterial wedge pressure and the ventricular stroke output. The results of blood serum examination were monitored. The main observed indexes included alanine aminotransferase, aspartate aminotransferase, creatinine, blood urea nitrogen, white blood cell count, blood platelet count, arterial oxygen saturation, arterial partial pressure of oxygen, arterial partial pressure of carbon dioxide and arterial power of hydrogen measured at seven time points: 24 hours before the operation (T1), 12 hours after resuscitation (T2), 24 hours after endotoxemia (T3), 72 hours after endotoxemia (T4), 96 hours after endotoxemia (T5), 144 hours after endotoxemia (T6), and 168 hours after endotoxemia (T7).

All of the experimental animals were given circulatory, respiratory and metabolic support. Dopamine was intravenously infused at a dose of 0.5 mg/kg/hour to maintain blood pressure when the heart rate was more than two times that of normal or the mean arterial pressure was less than 60% of normal. The animals were mechanically ventilated when the breathing rate was more than 40 breaths/minute or the arterial partial pressure of oxygen was below 60 mmHg. A solution of 5% glucose and 0.9% NaCl (100 to 150 ml/kg/day) (Baxter, Annapolis, Maryland, USA) with 10% KCl (1 ml/kg/day; Tianyi) was intravenously infused.

### Diagnostic criteria of MODS

The diagnostic criteria of MODS in experimental animals include the following: pulmonary dysfunction (breathing rate >40 breaths/minute, arterial partial pressure of oxygen <60 mmHg or arterial partial pressure of carbon dioxide >40 mmHg), cardiac dysfunction (cardiac dysrhythmia, heart rate more than two times the upper limit of normal, heart rate <60 beats/minute or mean arterial pressure <70% of the upper limit of normal), coagulation disorders (blood platelet count <70% of the upper limit of normal or the prothrombin time and thrombin time were 3 seconds longer than the upper limit of normal), hepatosis (serum alanine aminotransferase, serum aspartate aminotransferase or total bilirubin more than two times that of normal), and renal dysfunction (creatinine or blood urea nitrogen more than two times the upper limit of normal. MODS could be diagnosed if two or more criteria can be met.

### Flow cytometry studies

One hundred microliters of peripheral blood and 50 μl bone marrow in experimental animals at the seven time points described above were treated with 0.5 mM ethylenediaminetetraacetic acid (Sigma-Aldrich Chemie GmbH, Munich, Germany) as anticoagulant and were incubated for 30 minutes in the dark with activated protein C-labeled monoclonal rabbit KDR antibody (Lake Placid, New York, USA) and the phycoerythrin-labeled polyclonal goat CD133 antibody (Santa Cruz, California, USA). Isotype-identical antibodies IgG1-PE and IgG1-APC (Becton Dickinson, Franklin Lakes, New Jersey, USA) served as controls.

The analysis was carried out using a FACSCalibur flow cytometer with CellQuest software (BD Pharmingen, San Diego, California, USA). The cell surface expression of KDR was determined by flow kilometric analysis using 620 to 650 nm wavelength laser excitations and monitoring the emitted fluorescence with a detector optimized to collect peak emissions at 660 to 670 nm. The cell surface expression of CD133 was determined using 488/575 nm excitation and emission wavelengths.

### Endothelial progenitor cell culture assay

Mononuclear cells were isolated by density-gradient centrifugation with Ficoll (1.077 g/ml; Sigma) from 10 ml peripheral blood and 2 ml bone marrow in experimental animals at the seven time points (ethylenediamine tetraacetic acid as anticoagulant). Immediately after isolation, mononuclear cells were plated on six-well culture dishes coated with 2% human fibronectin (Chemicon, Billerica, Massachusetts, USA) at a density of 1 × 10^6^/cm^2 ^and were maintained in endothelial progenitor cell growth medium-2 (PromoCell, Heidelberg, Germany) for 2 hours. After 2 hours, nonadherent cells were collected and replated. After 4 days in culture, nonadherent cells were removed by a thorough washing with PBS. The culture was maintained through day 7, and adherent cells were subjected to further examinations.

### Characterization of endothelial progenitor cells

Immunohistochemical analysis for confirmation of the phenotype and fluorescent chemical detection of EPCs were performed on adherent mononuclear cells after 7 days in culture. Adherent mononuclear cells were stained with the following antibodies: monoclonal rabbit KDR antibody (Upstate) and polyclonal goat CD133 antibody (Santa Cruz). Direct fluorescent staining was used to detect dual binding of FITC-labeled Ulex europaeus agglutinin (UEA-1) (Sigma) and 1,1-dioctadecyl-3,3,3,3-tetramethylindocarbocyanine-labeled acetylated low-density lipoprotein (Dil-ac-LDL; Molecular Probe, Carlsbad, California, USA). Cells were first incubated with Dil-ac-LDL at 37°C and were later fixed with 2% paraformaldehyde (Tianyi) for 10 minutes. After being washed twice, the cells were reacted with UEA-1 (10 mg/l) for 1 hour. After the staining, samples were analyzed using a laser scanning confocal microscope (Leica, Wetzlar, Germany). Cells demonstrating double-positive fluorescence were identified as differentiating EPCs.

### Migratory and adhesive capacities of the endothelial progenitor cell assay

Isolated EPCs were detached using 1 mmol/l ethylenediamine tetraacetic acid in PBS (pH 7.4), were harvested by centrifugation, were resuspended in endothelial progenitor cell growth medium-2 and were counted. EPCs (1 × 10^4^) were added to in the upper chamber of a modified Boyden chamber with a polycarbonate filter (6.5 mm diameter, 8 μm pore size; Neuro Probe, Gaithersberg, Maryland, USA), and 500 μl endothelial progenitor cell growth medium-2 with human recombinant vascular endothelial growth factor (VEGF) (50 ng/ml) (Peprotech EC, London, UK) was added to the bottom chamber. After 24 hours of incubation at 37°C and 5% carbon dioxide, the lower side of the filter was washed with PBS and fixed with 2% paraformaldeyde. For quantification of migrated cells, cell nuclei were stained with Giemsa (Dade Behring, Marburg, Germany). Migrated cells in the lower chamber were counted manually in five random microscopic fields.

Fibronectin (100 μg/ml) was coated onto 96-well plates and left for 12 hours at 37°C. The first passages of EPCs at different time points were added into endothelial progenitor cell growth medium-2 at a density of 1 × 10^4^/ml. Then the medium was added into these plates with 1 ml/well and left to attach for 30 minutes. For quantification of migrated cells, cell nuclei were stained with Giemsa (Dade Behring). Attached cells were counted manually in five random microscopic fields.

### Angiogenesis assay

An angiogenesis assay plate (BD Pharmingen) was used to assay the angiogenetic capabilities of EPCs. The 96-well black plate (BD Pharmingen) with a clear bottom uniformly coated with BD Matrigel Matrix was allowed to polymerize for 30 minutes at 37°C and 5% carbon dioxide. The first passage of EPCs was added at different time points to the wells at a density of 1 × 10^5^/well. The angiogenesis assay plate was incubated for 24 hours at 37°C, 5% carbon dioxide. For each plate, 6.25 ml Hank's balanced saline solution (HBSS) (BD Pharmingen) was measured out and warmed to 37°C. We added 20 μl dimethylsulfoxide (BD Pharmingen) to each 50 μg vial of Calcein AM (8 μg/ml) solution (BD Pharmingen), and then added approximately 100 μl warm (37°C) HBSS to the vial. Following incubation, the medium was carefully removed from the plates. The plates were washed by adding 100 μl HBSS to each well, and the EPCs were then labeled by adding 50 μl/well of 8 μg/ml Calcein AM in HBSS and the plates were incubated for 30 minutes at 37°C, 5% carbon dioxide. The labeling solution was removed and the plates washed twice, and we then counted the tubes in the plate using a fluorescent microscope.

### Statistical analysis

Data are expressed as the mean ± standard deviation. Descriptive statistics were made on all test variables. A two-sample *t *test was used to compare the mean values of variables among the two groups of experimental animals, whereas the chi-square test was used to compare proportions on the normally distributed variables. A two-sample Wilcoxon rank sum test was used for variables that were not normally distributed. *P *< 0.05 was considered significant.

## Results

### Important organ function of animals and incidence of MODS

All of the animals in the experimental group presented MODS (100%) and 17 out of 20 animals (85%) died in the phase of observation. A total of 15 animals (75%) developed MODS in the midanaphase of the sepsis (69 to 144 hours post hemorrhagic shock + resuscitation + endotoxemia). In all of the animals with MODS, dysfunction of two organs occurred in seven cases, dysfunction of three organs occurred in eight cases and dysfunction of more than four organs occurred in five cases. The incidence of MODS and death of the animals in the control group were 0% (*P *< 0.01).

In the experimental group, the incidence of pulmonary dysfunction was the 80% (16 cases), the incidence of cardiac dysfunction was 65% (13 cases), the incidence of hepatosis was 55% (11 cases), the incidence of renal dysfunction was 35% (seven cases) and the incidence of coagulation disorders was 35% (seven cases). Pulmonary dysfunction and cardiac dysfunction occurred earlier than any other organ dysfunction in the course of sepsis. The breath rate began to increase and the oxygen pressure (PO_2_) began to decrease sharply at 72 hours after endotoxemia (T4) and continually increased to peak at 144 hours after endotoxemia (T6). The values of the heart rate, the MAP, alanine aminotransferase, total bilirubin, the prothrombin time, serum creatinine and serum blood urea nitrogen had similar changes. The values of the blood platelet count began to decrease at T4 and decreased sharply to the bottom at T5. All of the values of the survival animals normalized gradually during 120 to 144 hours after endotoxemia.

### Number of endothelial progenitor cells in the progression of MODS

**Figure 1:**
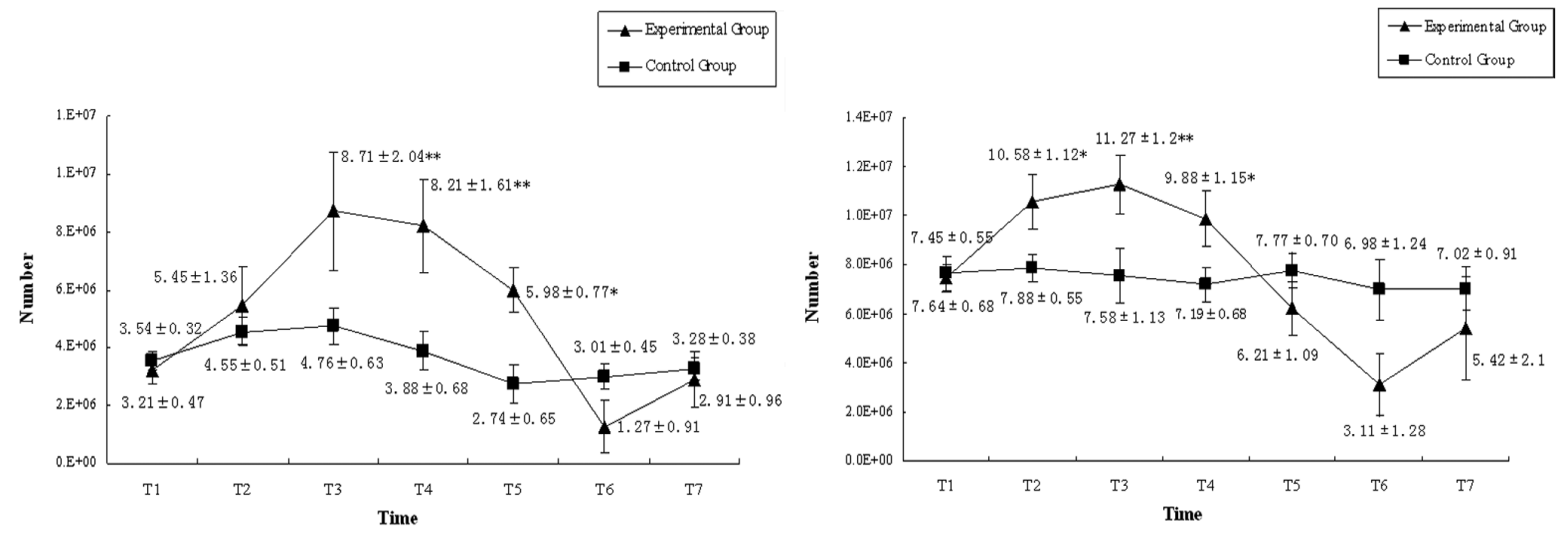
Number of endothelial progenitor cells during progression of multiple organ dysfunction syndromes. The number of endothelial progenitor cells in (left) peripheral blood and (right) bone marrow. *P < 0.05, **P < 0.01.

The number of EPCs in peripheral circulation and bone marrow decreased sharply in the progression of MODS. In the experimental group, the number of EPCs in peripheral circulation increased after hemorrhagic shock and continually increased to peak in the earlier phase of sepsis (72 hours after endotoxemia). With the progression of MODS the number of EPCs would decrease sharply until death, but the number of EPCs in surviving animals would begin to increase at the 144th hour after endotoxemia. Similar changes of EPCs in peripheral circulation were also found in bone-marrow-derived EPCs, but the magnitude of changes was greater and the timing point of the increase was earlier (24 hours after endotoxemia). In the control group, the number of EPCs in peripheral circulation and in the bone marrow increased a little after operation but quickly returned to normal (Figure 1).

### Endothelial progenitor cell culture assay

**Figure 2:**
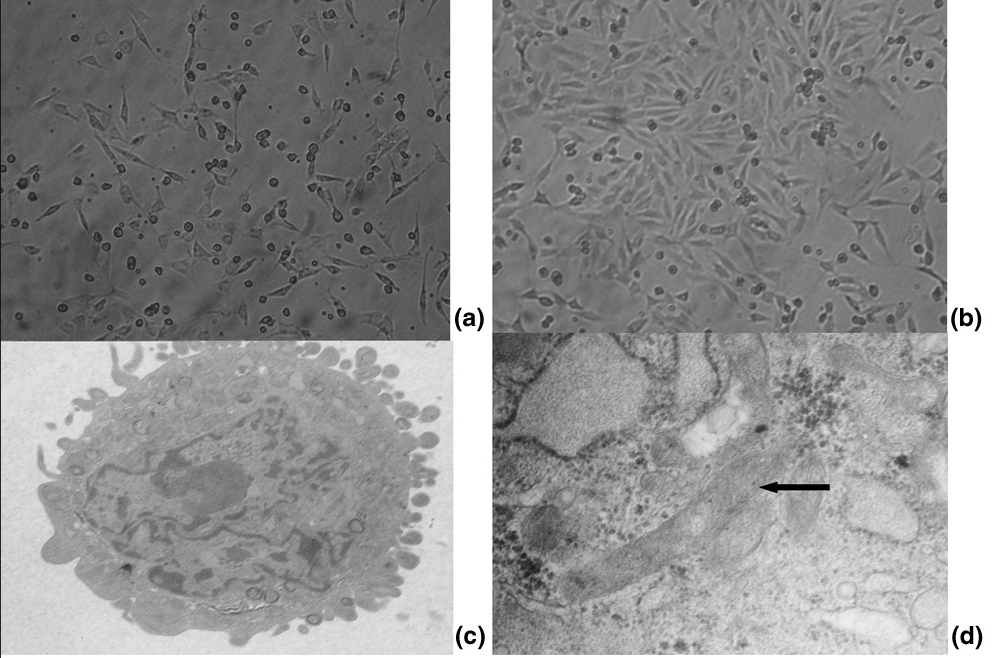
Endothelial progenitor cell morphology during *ex vivo *culture. **(a) **Mononuclear cells are able to differentiate into spindle cells 48 hours after culture.**(b) **Colony of endothelial progenitor cells (EPCs) observed after 7 or 8 days of culture. **(c) **and **(d) **After 7 days of culture, the ultrastructure of EPCs can be observed by electron microscope. Black arrow, Weible-Palade body that was the characteristic structure of EPCs. Magnification: (a) and (b) ×100; (c) ×5,000; (d) ×30,000.

The mononuclear cells were round or oval and none was attached at the first day of culture. Alignments of spindle cells were observed after 3 days of culture. After 7 days of culture, some of the EPCs formed a cobblestone-like structure and took part in network formation. Some rod-shaped organelles (Weible-Palade bodies) that were deemed the characteristic structure of EPCs could be observed in cell plasma by transmission electron microscopy (Figure 2).

### Characterization of endothelial progenitor cells

**Figure 3:**
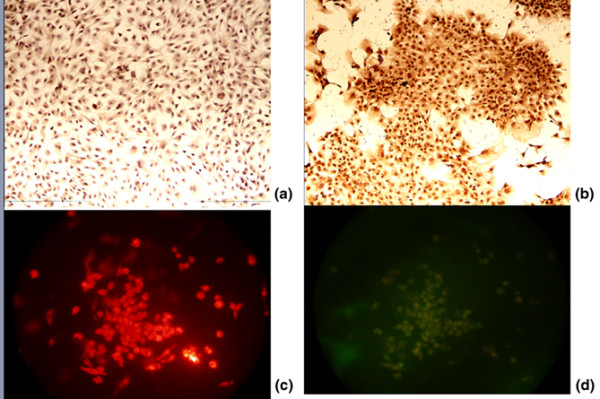
Characterization of endothelial progenitor cells. At 7 days of culture, immunohistochemical staining revealed that more than 90% of the cultured cells are positive to **(a) **CD133 and **(b) **vascular endothelial growth factor receptor-2. Endothelial progenitor cells were incubated with 1,1-dioctadecyl-3,3,3,3-tetramethylindocarbocyanine-labeled acetylated low-density lipoprotein (Dil-Ac-LDL) and stained with Ulex europaeus agglutinin (UEA-1). Fluorescence microscopy illustrates that endothelial progenitor cells are positive for **(c) **Dil-Ac-LDL and **(d) **Dil-Ac-LDL and UEA-1. Magnification: ×100.

The attached spindle cells at different points were all positive for taking up Dil-ac-LDL and UEA-1. Co-staining cells revealed that more than 90% of adherent cells are both Dil-ac-LDL-positive and UEA-1-positive. Additional staining revealed that more than 90% of the cultured cells were positive for KDR and CD133 (Figure 3).

### Migratory and adhesive capacities of endothelial progenitor cells in the progression of MODS

**Figure 4:**
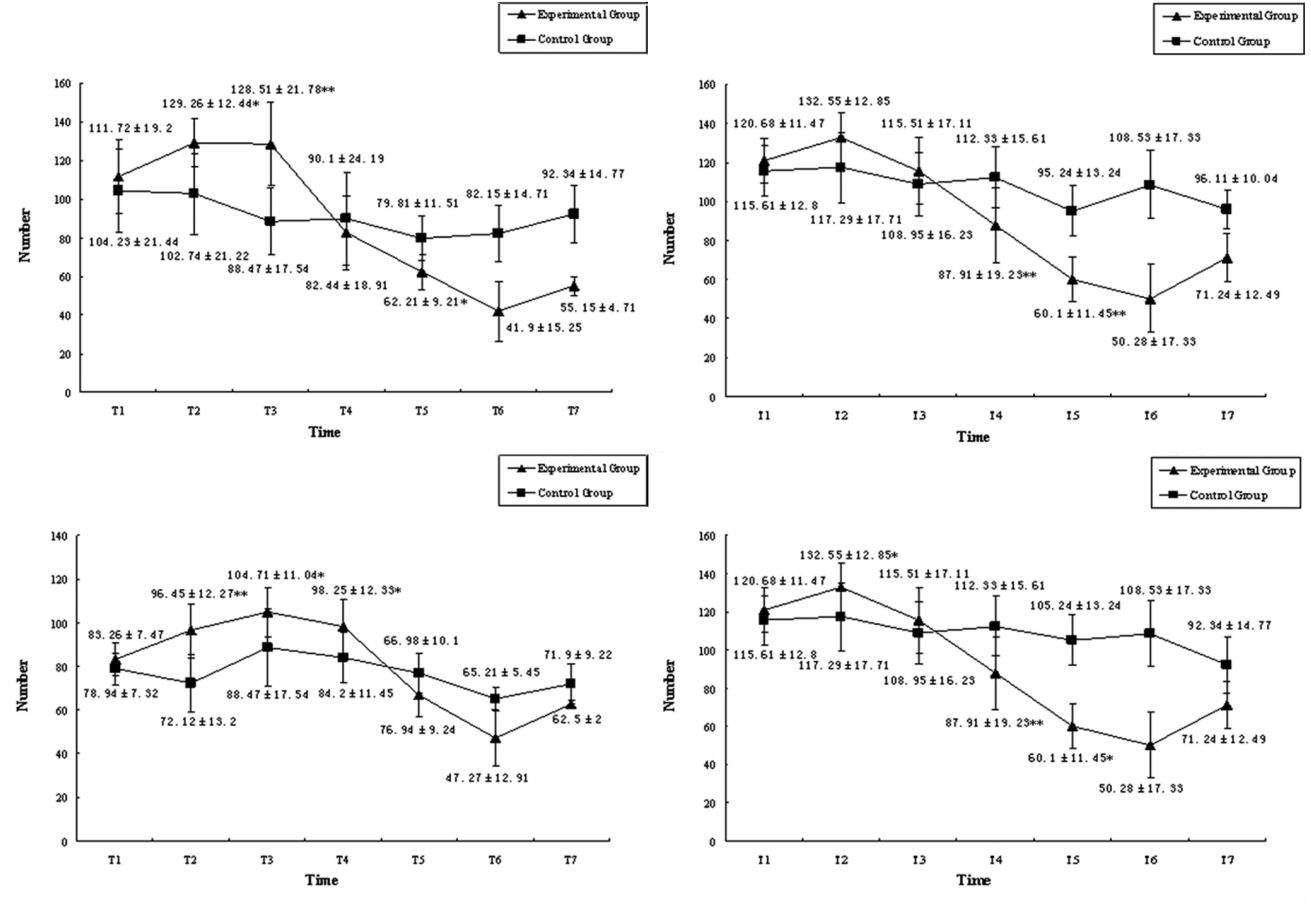
Migratory and adhesive capacities of endothelial progenitor cells during progression of multiple organ dysfunction syndromes. The (top) migratory capacities and (bottom) adhesive capacities of endothelial progenitor cells in (left) peripheral blood and (right) bone marrow. *P < 0.05, **P < 0.01.

In the experimental group, the migratory and adhesive capacities of EPCs in both peripheral circulation and the bone marrow increased quickly after hemorrhagic shock and continually increased to peak in the earlier phase of sepsis (24 hours after endotoxemia). With the progression of MODS the migratory and adhesive capacities of EPCs would decrease quickly and sharply until death, but they would begin to increase at the 120th hour after endotoxemia in the surviving animals. Similar changes of migratory and adhesive capacities of EPCs in peripheral circulation were also found in the EPCs from bone marrow. Moreover, the time point for the decrease of migratory and adhesive function was earlier than that of the number of EPCs. In the control group, the migratory and adhesive function of EPCs in both peripheral circulation and the bone marrow increased a little after operation but quickly returned to normal (Figure 4).

### Angiogenesis assay

**Figure 5:**
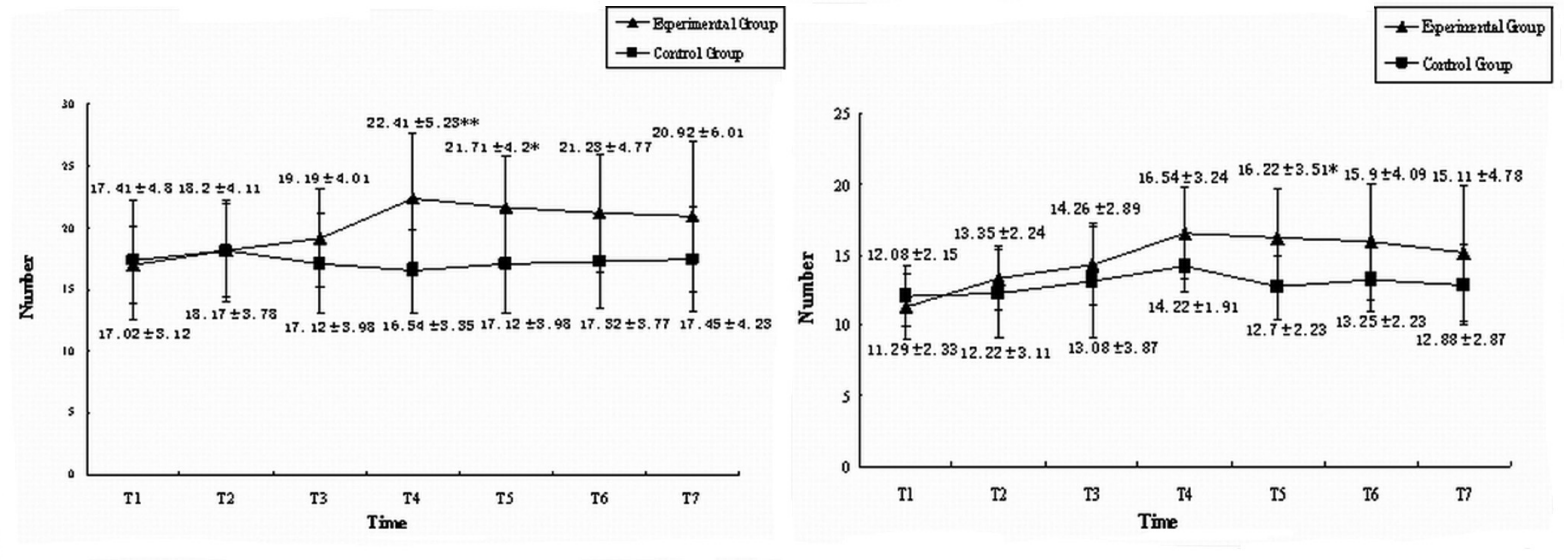
Angiogenic capacity of endothelial progenitor cells during progression of multiple organ dysfunction syndromes. The angiogenic capacity of endothelial progenitor cells in (left) peripheral blood and (right) bone marrow. *P < 0.05, **P < 0.01.

**Figure 6:**
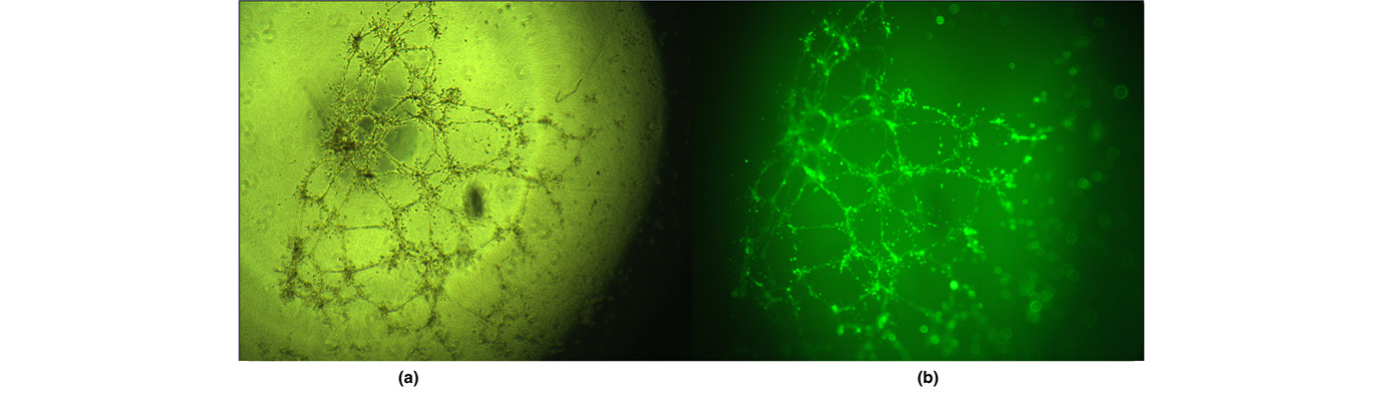
Angiogenesis function of endothelial progenitor cells. Angiogenesis function of endothelial progenitor cells with Calcein added, observed by **(a)** phase-contrast microscope and **(b)** fluorescent microscope. Magnification: ×400.

The number of tubes in the plate was used to illustrate the angiogenesis function of EPCs at different time points. The results showed that the angiogenesis function of EPCs from peripheral circulation was stronger than that from bone marrow. In the experimental group, the angiogenesis function of EPCs in both peripheral circulation and the bone marrow would increase gradually after hemorrhagic shock and continually increased to peak in the metaphase of sepsis (72 hours after endotoxemia). The angiogenesis function of EPCs in both peripheral circulation and the bone marrow would decrease gradually with the progression of MODS, but was still stronger than normal. In the control group, the angiogenesis function of EPCs in peripheral circulation and the bone marrow maintained normal status without much change (Figures 5 and 6).

## Discussion

The present study observed for the first time that the number and function of EPCs decreased sharply in the progression of MODS, and the decrease was prior to the occurrence of MODS in the experimental animals. There have been several studies focused on the relation between EPCs and sepsis [8-10], but no systematic studies existed regarding pathological variations in the number and function of EPCs *in vivo *in MODS and there was no agreement in the change of EPCs in MODS caused by severe sepsis.

Mayr and colleagues detected that EPCs would decrease to a nadir 6 hours after infusion of sepsis and would return to values comparable with baseline 24 hours after lipopolysaccharide of coli bacillus challenge [10], and conceived that the 200-fold increase in TNF outweighed the comparably moderate increases in VEGF and granulocyte colony-stimulating factor (G-CSF) that finally resulted in a net EPC decrease. We suggested, however, that such a low-grade endotoxemia model may not be deemed a sepsis model, which may partly explain the different results between our experiment. Rafat and colleagues [9] found that the circulation endothelial progenitor cells (CEPCs) increased from the 6th hour after diagnosis as sepsis and remained high during the sepsis phase, but none had significantly low numbers of EPCs [9]. In our experiment, all of the animals in the experimental group presented MODS and most of them died. The results in the nonsurviving animals were similar to the study of Rafat and colleagues [9] but the EPCs of surviving animals decreased sharply in the progression of MODS and began to increase in the 144th hour after endotoxemia, which may be caused by more severe sepsis. This may have caused the difference between our two studies.

Our study lends further support to the previous observations that the EPC number and function decreases with advancing sepsis. We also deemed that the pool of EPCs would be exhausted and the repair capacity would be impaired by severe trauma and inflammation, which would accelerate the progression of MODS. We therefore postulate that autologous transplantation of EPCs may play an important role in the prevention and treatment of MODS.

The pathogenesis of MODS was so complicated that we did not investigate the correlation between the number and capacity of EPCs and the other factors of MODS in the present study. The mechanisms for the decrease in the number and function of EPCs in the progression of MODS still remain to be determined. Another limitation of the present study was that the functional activity of EPCs was tested *in vitro*, and the results of this analysis may not necessarily correlate well with the response *in vivo*. Whether EPCs could differentiate to mature ECs *in vivo *to prevent the progression of MODS requires further investigation.

In the present study, the level of EPCs in peripheral circulation and in the bone marrow was directly quantified by flow cytometry measurement of the percentage of CD133/KDR double-positive mononuclear cells. There is still controversy about which markers should be used to characterize the EPCs. According to recent studies [11,12], EPCs were defined as cells positive for both hematopoietic stem cell markers and endothelial markers, such as CD133, CD34 and KDR. CD133 and CD34 were deemed the two main hematopoietic stem cell markers. Unlike CD34, however, CD133 was not expressed on mature ECs [9]. CD133/KDR double-positive cells could therefore more probably reflect EPCs.

The results demonstrate that the number of EPCs and the migratory and adhesive capacities of EPCs increased quickly after hemorrhagic shock and continually increased to peak in the earlier phase of sepsis. With the progression of MODS, these factors would decrease sharply until death; and the decrease of migratory and adhesive capacities of EPCs was prior to the decrease of the number of EPCs. The angiogenesis function of EPCs from peripheral circulation was stronger than that from bone marrow and would change gradually after injuries. The angiogenesis function would, however, still be stronger than normal even until death. The results showed that the occurrence of the decrease of the number and function of EPCs was prior to the occurrence of MODS, so the sharp decrease in the number and function of EPCs in the progression of MODS may be one of the main pathogenic factors of MODS. In the present study, the migratory, adhesive and angiogenesis capacities of EPCs were detected on the EPCs that had been cultured for 7 days *in vitro *because we could not isolate EPCs directly. We found that these functions of EPCs would remain stable after short-time culturation. The functions of EPCs were therefore deemed able to reflect the functions of EPCs *in vivo*.

The unique angiogenic capacity of EPCs renders them optimal candidates for cell-based therapies. Recent studies have described that both peripheral circulation and the bone marrow can be used as a source of EPCs, which have the potential to differentiate into functional ECs under specific culture conditions [12]. The methods in previous studies of purifying and culturing EPCs had always relied on magnetic bead or cytofluorometric selection for cells expressing CD34, KDR, or CD133 [13,14]. In our research we showed that EPCs could be isolated from peripheral circulation and the bone marrow based on their adherence and requirement for specific growth conditions as ECs without any further enrichment steps. We also identified cultured EPCs by taking up Dilac-LDL as well as UEA-1 and by other phenotype confirmation. Our results showed that more than 90% of the adherent cells could take up both Dil-ac-LDL and UEA-1, and KDR and CD133 positive. The method of isolation and culture of EPCs is therefore feasible and may provide adequate cells for cell-based vasculogenesis therapy.

Another important finding was that the angiogenic capacity of EPCs in peripheral circulation was stronger than that of the EPCs from bone marrow and remained stable in the progression of MODS. The capacity indicated that EPCs in peripheral circulation had changed after being mobilized from the bone marrow. In recent studies two different EPC subpopulations have been described, denoted as early EPCs and late EPCs, with distinct cell growth patterns and ability to secrete angiogenic factors [15,16]. Early EPCs are spindle-shaped cells, including the EPCs in the bone marrow and the EPCs just mobilized to peripheral circulation [17]. Late EPCs are cobblestone shaped, including the EPCs mobilized to peripheral circulation and precursors of mature ECs [17,18]. Our results show that late EPCs had stronger angiogenic capacity than that of early EPCs; therefore, late EPCs could play a more important role in angiogenesis in cell-based therapy for ischemic diseases.

## Conclusions

The present study demonstrates, for the first time, the change in number and in function of EPCs in peripheral circulation and in the bone marrow in MODS, and found that the number and function of EPCs would decrease in the progression of MODS and may be one of the main pathogenic factors of MODS. The results of this study may therefore be extended to more clinical implications, but further prospective studies are needed to evaluate whether the level of EPCs can serve as a valuable biological marker and can be used for the stratification of patients with MODS.

## Key messages

• The number and function of EPCs decreased sharply in the progression of severe sepsis, prior to the occurrence of MODS.

• The decrease in the number and function of EPCs may be one of main pathogenic factors of MODS.

• The angiogenic capacity of EPCs in peripheral circulation was stronger than that of the EPCs from bone marrow and remained stable in the progression of MODS.

## Abbreviations

CEPC: circulation endothelial progenitor cell; Dil-ac-LDL: 1,1-dioctadecyl-3,3,3,3-tetramethylindocarbocyanine-labeled acetylated low-density lipoprotein; EC: endothelial cell; EPC: endothelial progenitor cell; G-CSF: granulocyte colony-stimulating factor; HBSS: Hank's balanced saline solution; KDR: vascular endothelial growth factor receptor-2; MAP: mean arterial blood pressure; MODS: multiple organ dysfunction syndromes; PBS: phosphate-buffered saline; PO_2_: oxygen pressure; UEA-1: Ulex europaeus agglutinin; VEGF: vascular endothelial growth factor.

## Competing interests

The present study is funded by the China National Foundation of Natural Science (30672170).

## Authors' contributions

THL, YW and ZML established the model of experimental animals. THL, YW, ZML and HZ carried out the molecular genetic studies and culture of EPCs. TML and YW drafted the manuscript. JWB, LYM and XCX carried out the culture and the assay of EPCs. GEF conceived of the study, and participated in its design and coordination. All authors read and approved the final manuscript.
